# Space Ultrasound: A Proposal for Competency-based Ultrasound Training for In-flight Space Medicine

**DOI:** 10.5811/westjem.18422

**Published:** 2024-02-13

**Authors:** Chanel Fischetti, Emily Frisch, Michael Loesche, Andrew Goldsmith, Ben Mormann, Joseph S. Savage, Roger Dias, Nicole Duggan

**Affiliations:** * Brigham and Women’s Hospital, Harvard Medical School, Department of Emergency Medicine, Boston, Massachusetts; † Cleveland Clinic, Department of Obstetrics and Gynecology, Cleveland, Ohio; ‡ Yale Bridgeport Hospital, Department of Emergency Medicine, Bridgeport, Connecticut

## Abstract

Space travel has transformed in the past several years. Given the burgeoning market for space tourism, in-flight medical emergencies are likely to be expected. Ultrasound is one of the few diagnostic and therapeutic modalities available for astronauts in space. However, while point-of-care ultrasound (POCUS) is available, there is no current standard of training for astronaut preparation. We suggest an organized and structured methodology by which astronauts should best prepare for space with the medical equipment available on board. As technology continues to evolve, the assistance of other artificial intelligence and augmented reality systems are likely to facilitate training and dynamic real-time needs during space emergencies.

**Summary:**

As space tourism continues to evolve, an organized methodology for POCUS use is advised to best prepare astronauts for space.

## BACKGROUND

Over the last decade, impressive technological advances surrounding space travel have made space tourism a reality for the not-too-distant future. As commercial industry increasingly lays down a stake in this nascent market, prior barriers to private-passenger space travel, such as cost and accessibility, are rapidly falling.^1^ ^,^ ^2^ Proposed opportunities for private passengers range from long-haul global travel through space, to brief orbit, to prolonged stay with hotel accommodations.^3^ As of late 2021, less than a handful of the nearly 600 individuals who have gone to space were civilian passengers.^4^ Despite small numbers currently, it is projected that by 2030 both space tourism and long-haul travel by space will capture nearly $20 billion of the larger space economy.^4^

Professional astronauts are often screened for baseline health conditions that could lead to in-flight medical emergencies and potentially jeopardize personnel safety or the mission.^5^ Therefore, true in-flight medical emergencies to date have been rare. However, with the greater diversity of traveler anatomy, physiology, and medical history, which will inevitably result from expansion of private-passenger space travel, a significant increase in in-flight medical emergencies is expected.^1^ ^,^ ^2^ ^,^ ^5^ For longer duration missions, it is projected that at least one medical emergency will occur per crew of six travelers.^5^ Unlike for medical emergencies during air travel, emergency landings and real-time conversations with ground control are not reliable options in space.^6^ ^,^ ^7^ Additionally, as more flights depart it will be increasingly unlikely that a trained medical doctor will be available or present on each flight. In fact, SpaceX just recently launched an all-civilian mission crew with only a trained physician assistant.^8^ Thus, with the expansion of the private space flight industry, innovative medical protocols and approaches must be developed.^9^

## OBJECTIVES

### Point-of-care Ultrasound Training for Space Medicine

#### Current Training Standards

Prior to current space travel, flight crews are required to train for anticipated mechanical, mission, and engineering challenges.^3^ Medical care is the responsibility of the crew medical officer (CMO) who typically has limited prior medical knowledge.^10^ The CMO training involves 40–80 hours of hands-on training with remediation and continuing virtual trainings as needed.^10^ Some of this preparatory training includes rudimentary medical education (phlebotomy, vital sign measurement, tonopen use, panoptic use, and ultrasound)^11^ designed in anticipation of coordinated care with Mission Control for telehealth interpretations.^11^ This often consists of “just-in-time” diagnostic algorithms to facilitate ultrasound interpretation with the aid of live telehealth guidance.^10^ Flight surgeons are frequently and regularly on console at Flight Control Room 1-Mission Control and actively participate in medical monitoring and guidance.

Point-of-care ultrasound (POCUS) images can be downloaded in real time for evaluation, and by using a private medical conference channel loop, only the ultrasound operator (trained and under non-disclosure agreement), the physician, and the patient/subject are involved. Even the mission’s flight director would not have access. Inherently, telemedicine has been a part of the International Space Station (ISS) since it launched. As longer space duration missions and interplanetary travel progress, time lapses of 40 minutes or longer are anticipated for ground crew virtual contact.^6^ ^,^ ^7^ ^,^ ^12^ ^,^ ^13^ These communication delays could lead to severe medical consequences for missions with flight crew trained according to the current standard of care.^6^ ^,^ ^7^ ^,^ ^12^

Because of its portability, low-cost, and radiation-free, real-time imaging for an impressive array of medical conditions, POCUS has a demonstrated utility in space medicine, In cases where ultrasound training is currently provided, a maximum of 2-3 hours is allotted throughout the entire pre-flight training curriculum.^11^ ^,^ ^14^ This Advanced Diagnostic Ultrasound in Microgravity (ADUM) educational program is used on the ISS where “cue cards” are used to rapidly guide non-expert users to perform ultrasounds on patients, with more than 90% accuracy after just minutes of training.^14^ While “cue cards” can be used, ADUM has found that non-medical operators can obtain quality data with the right amount of training and direction.^14^ For this reason, an on-board proficiency enhancement has also been created both in English and Russian.^14^ The combination of this several-hour training course with the “remote expert guidance” (available by Mission Control) is the most effective means by which in-flight ultrasound guidance is currently conducted with attention to limitations of ultrasound in space (gel use, device battery life, etc).^14^

In contrast, true mastery of POCUS for healthcare professionals typically requires years of practice during medical residency and often an additional year of dedicated training through an ultrasound fellowship. While mastery of POCUS at the same level of a medical professional is not realistic for most flight surgeon training, introducing POCUS to crew members and a flight surgeon’s repertoire through a structured and systematic curriculum has the potential to yield significant benefit to both private passengers and potentially the entirety of the mission. Additionally, in longer duration flight missions when emergency decisions need to be made using ADUM’s proposed telecommunication and ultrasound video transmission, time and video delays have real and significant limitations for astronaut care and outcomes.

## CURRICULAR DESIGN

### Proposed Point-of-care Ultrasound Training Solutions

Prior data on POCUS education suggests that even novice POCUS learners can retain the basics of image acquisition and interpretation with a minimum amount of focused training.^14^ ^–^ ^18^ Core competency in scanning each organ system can be achieved with a two-hour session of combined didactics and hands-on scanning.^19^ ^–^ ^22^ Thus, as a consortium of medical doctors and experts, we propose a structured, competency-based POCUS curriculum for commercial space travel that includes well-defined aims targeting image acquisition and interpretation for the most common organ systems involved in in-flight medical emergencies (Figure 1A). Astronauts trained for space should be considered technicians in these scenarios, with physicians supplementing the real-time diagnoses and treatments.

**Figure 1A.**
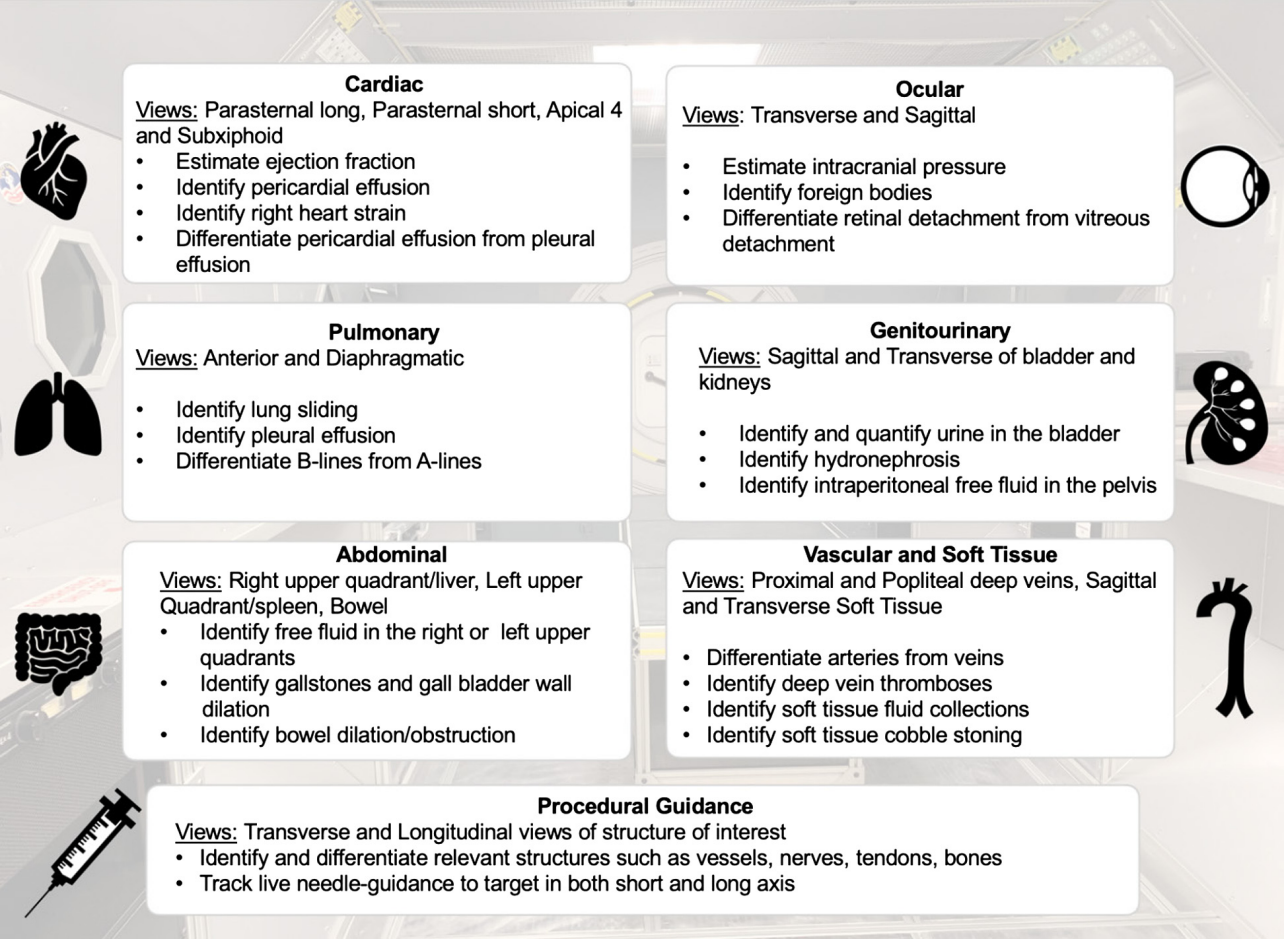
Core point-of-care ultrasound competencies in a structured ultrasound training program.

The seven most high-yield procedure or organ-based systems are identified with an advised 1–2 hours training per topic. Realistically, a one-day course of about 6–8 hours would be sufficient to satisfy a foundation for competency. However, as pre-mission astronaut preparation time is busy and filled with requirements, these preparatory courses can be adjusted and elongated as tolerated by individual mission schedules and needs. This structured format would ensure consistent and homogenous training for all astronauts anticipated in space. Each aspect of the mission is rehearsed, and each astronaut (and back-up astronaut) is also cross-trained for activities outside their primary mission designation scope, in the event of astronaut drop-out. Training would be mission-specific and expected to be intensive and start about three years before launch. Real-time updates can be made if mission requirements change at any point within the three years to launch, so that the most up-to-date equipment and procedures are used prior to launch.

Core competency is an appropriate goal for most flight surgeons in training and should include the basic skills needed for POCUS image acquisition and a proficient level of independent interpretation. Although far from mastery level, core competency allows for an appropriate balance of limited input of training time required. The skills of POCUS acquisition and interpretation can always be supplemented with adjuvant tools such as live telehealth with ground control, or (artificial reality/artificial intelligence [AI]) tools during live missions (Figure 1B). For travel at lower altitudes of orbit, lower tiered competency coupled with available telehealth guidance may be sufficient.^23^ ^–^ ^25^ For long-haul or deeper space travel, however, completing the entire curriculum, perhaps with progression to advanced-level training, is highly recommended. For any level of training, learning can be consolidated by remediation and spaced repetition of training through augmented reality and recorded lectures.

**Figure 1B.**
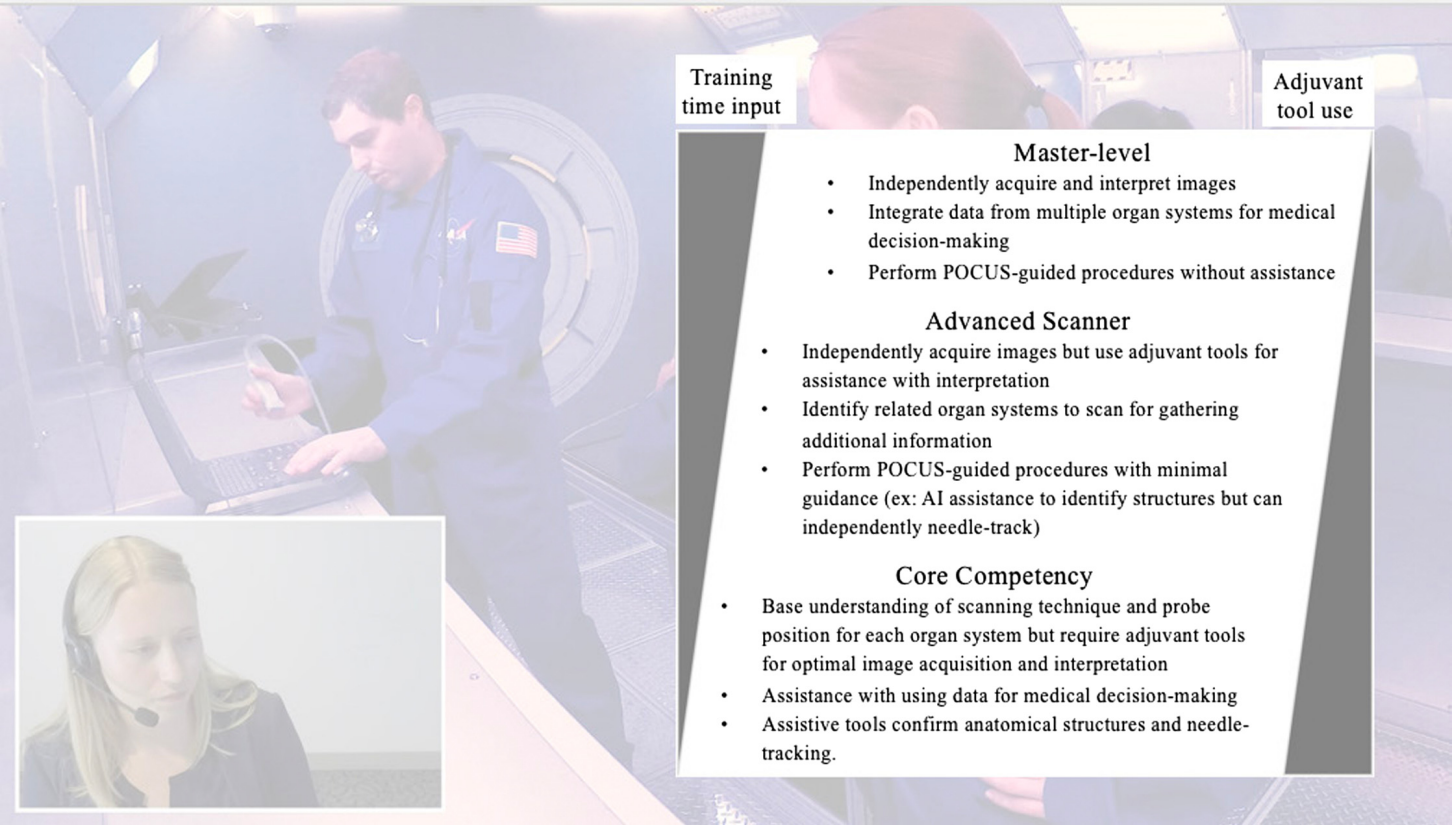
Suggested tiered competency-based ultrasound training. Images from the STRATUS Space Simulation training.

## IMPACT AND EFFECTIVENESS

### Point-of-care Ultrasound and Space Medicine

While POCUS has been used by the National and Aeronautics Space Administration (NASA) as the primary form of imaging aboard the ISS since 1982, original devices offered only rudimentary imaging capabilities.^12^ ^,^ ^27^ ^–^ ^29^ Today POCUS devices are capable of advanced imaging with multiple frequencies and modalities for both diagnostic and therapeutic applications.^30^ Many POCUS devices are now hand-held, which offers a unique advantage over alternative imaging modalities in settings where weight and volume restrictions are critical, such as in space travel.^6^ ^–^ ^8^ Consequently, POCUS represents an ideal imaging modality for the growing space medicine industry.^8^

Previously described medical emergencies in space span nearly all organ systems and reflect the unique physiological stress placed on the human body by microgravity and other natural risks in space such as dehydration (Table 1).^5^ ^,^ ^9^ ^,^ ^31^ Similarly, there are scenarios in zero gravity, such as scanning for free fluid for trauma (as in cases with focused assessment with sonography for trauma exams) or for pleural edema (pneumonia or other infectious or cardiac conditions), that have alternative interpretations given the gravity-less conditions. On Earth, blood or fluid would pool in certain areas of the body (the bladder recess or inferior aspects of the lungs), but in zero gravity, there is no proclivity for pooling in any one specific area; hence, a complete and thorough exam is important to train for. As demonstrated in Table 1, POCUS has a potential role in assessing medical conditions associated with nearly every organ system in space travel including cardiac, pulmonary, genitourinary, and ocular complaints. Although expansive, this list does not address the array of potential POCUS-guided critical procedures. Anticipated in-flight procedures include the following: establishing vascular access; regional anesthesia for acute pain control (or rare but life-saving procedures); and pericardiocentesis and needle decompression for tension pneumothorax. Additionally, there are many other important implications for the preparation of POCUS use in space and adjustments that must be made and trained for in zero-gravity conditions. For example, ultrasound gel is not used in space, in part because water is equally as effective and because duplicate use of items is critical for the cost and weight restrictions imposed for each launch.

**Table 1.** Medical emergencies with respective incidences in space and related utility of point-of-care ultrasound.

Physiological system	Medical events	Incidence in space (% reported)	Pathophysiology	Ultrasound indication/ POCUS finding
Ocular and sensory organs	• Ocular foreign body • Increased intracranial pressure • Disequilibrium	Up to 42%^32^	Foreign bodies from exposures within the space capsule or orbit	• Identify ocular foreign bodies • Measure optic nerve sheath diameter • Measure optic nerve sheath diameter
Cardiac	Arrhythmias	0.2–9.55%	Shifting fluids and dynamic changes in gravitational movements can cause compensatory changes in both pulmonary and cardiac volumes and potentially provoking cardiac arrhythmias and cardiac irritability^33^ ^,^ ^34^	Transthoracic echo for arrhythmias, wall motion abnormalities, or cardiac standstill
	Pulmonary embolism	***	Lack of gravity and venous stasis in space can promote thrombotic events	Transthoracic echo for right ventricular strain
Pulmonary	• Pneumothorax • Respiratory infections	7.6–64%^35^	Barotrauma	Confirm lung sliding
			Dysregulation of the immune system with possible concurrent viral reactivation^36^	Identify pulmonary B-lines or consolidations
Vascular	Venous thromboembolism	*** ^37^ ^,^ ^38^	Lack of gravity and venous stasis in space can promote thrombotic events^31^	Identify deep vein thromboses
Gastrointestinal	Bowel obstruction and constipation	*** ^39^	Constipation is common in space but symptoms can mimic bowel obstruction	Abdominal POCUS for bowel obstruction
Genitourinary	• Acute urinary retention • Renal stones	∼1.20%^32^	Often multifactorial, pharmacologic, loss of gravitational forces, and demanding schedules with limited access to voiding are considered contributors to urinary retention^40^	Measure post-void bladder volume
		0–5%	Bone loss and muscle wasting can lead to increased calcium excretion that can precipitate renal stones^41^	Identify hydronephrosis
Dermatological	Soft tissue infections	8–10%^32^	In a gravity-less environment, bacteria and other pathologic flora can potentially linger longer on the skin’s surface	Confirm abscess vs cellulitis
Traumatic injuries	• Intra-abdominal bleeding • Fractures • Joint injuries • Soft tissue injuries	11–26%^32^	Trauma^27^	• eFAST for intraperitoneal free fluid • Identify bony abnormalities • Identify joint effusions • Identify hematomas, etc

*** Indicates described reports of pathophysiology in space without disclosed numerical values in space or with little to no episodes in space. Terrestrial incidences are often used for risk stratification modeling.

*POCUS*, point-of-care ultrasound; *eFAST*, extended focused assessment with sonography for trauma.

## CONCLUSION

### Future of Point-of-care Ultrasound in Space Medicine

While there are a variety of ultrasound applications not described here, the identified organ systems listed were chosen based on frequency of emergencies and the anticipation of in-flight medical needs.^42^ While these recommendations have yet to be tested and applied for space medicine practices, based on similar POCUS education models, skill retention is likely to be high among astronauts.^11^ ^,^ ^23^ Spaced repetition and remediation will help consolidate skills and can be instrumental in maintaining fluency long term.^23^ ^,^ ^26^ Alternative learning modalities such as virtual reality and mixed in-person training modules can assist with skill retention in real time when live tele-consults are unavailable. Similarly, AI algorithms have the potential to offer automated image interpretation and clinical-decision assistance without the need for live tele-support.

Implementing a structured POCUS curriculum has the potential to make tangible changes to in-flight healthcare and emergency procedures, which will be crucial as the space flight industry continues to evolve. To maximize the utility of this diagnostic and therapeutic device, we propose that POCUS education should be a prerequisite of training for space flight for both near-future and future missions and can be achieved through a structured curriculum to make the most efficient use of astronaut training and time.^11^ ^,^ ^29^ ^,^ ^43^ ^,^ ^44^ While time allocation is an exceptionally valuable resource in astronaut training and education, integrating POCUS education into the mandatory space-flight training curriculum will likely pay off in dividends for future passengers and missions.
